# Pathogenic Mechanisms and Therapeutic Approaches in Obesity-Related Knee Osteoarthritis

**DOI:** 10.3390/biomedicines12010009

**Published:** 2023-12-20

**Authors:** Russka Shumnalieva, Georgi Kotov, Plamena Ermencheva, Simeon Monov

**Affiliations:** 1 Department of Rheumatology, Medical University of Sofia, 1431 Sofia, Bulgaria; rshumnalieva@yahoo.com (R.S.); doctor_monov@yahoo.com (S.M.); 2 Clinic of Rheumatology, University Hospital ‘St. Ivan Rilski’, 1612 Sofia, Bulgaria; p.ermencheva@gmail.com

**Keywords:** osteoarthritis, pain, cartilage degeneration, molecular pain pathway

## Abstract

The knee is the joint most frequently involved in osteoarthritis, a common joint disorder in the adult population that is associated with significant chronic joint pain, reduced mobility and quality of life. Recent studies have established an association between obesity and the development of knee osteoarthritis that goes beyond the increased mechanical load on the knees as weight-bearing joints. This link is based on the maintenance of a chronic low-grade inflammation, altered secretion of adipokines by the adipose tissue and development of sarcopenia. Major adipokines involved in the pathogenesis of obesity-related knee osteoarthritis include adiponectin, which appears to have a protective effect, as well as leptin, resistin and visfatin, which are associated with higher pain scores and more severe structural damage. Joint pain in knee osteoarthritis may be both nociceptive and neuropathic and is the result of complex mechanisms driven by nerve growth factor, calcitonin gene-related peptide and pro-inflammatory cytokines. The role of endogenous cannabinoids and gut microbiota in common mechanisms between obesity and knee pain has recently been studied. The aim of the present review is to highlight major pathogenic mechanisms in obesity-related knee osteoarthritis with special attention on pain and to comment on possible therapeutic approaches.

## 1. Introduction

Osteoarthritis (OA) is a common joint disorder in the adult population and is widely associated with chronic joint pain, reduced mobility and impaired productivity [1,2,3]. The knee is the most commonly affected joint, followed by the interphalangeal joints of the hand and the hip [4]. The development of OA is related to a plethora of factors such as age, sex, genetics, trauma and increased mechanical load, as well as various concomitant diseases. In particular, obesity-related OA, also referred to as metabolic syndrome-associated OA, has recently been recognized as a separate entity [2,3,5]. It has been shown that obesity is related to knee OA not simply through an increased mechanical load on weight-bearing joints but through the maintenance of chronic inflammation, altered secretion of adipokines and development of sarcopenia [2,3]. In addition, a higher degree of obesity has been associated with a higher pain score [3]. Recent recommendations on the therapeutic management of knee OA, however, do not include specific guidelines in cases of obesity-related knee OA [6]. Therefore, the aim of this review was to outline the key pathogenic aspects in obesity-related knee OA and to present current understanding of pain mechanisms and therapeutic approaches while commenting, where possible, on those that may be beneficial in knee OA associated with metabolic syndrome.

## 2. Joint Alterations in Knee OA

Knee OA is a chronic degenerative joint disease that not only involves the joint cartilage and the underlying subchondral bone, but also the synovial membrane, adjacent muscles and ligaments, as well as Hoffa’s fat pad in a pattern that has been termed ‘whole joint disease’ [7,8].

Articular cartilage is a connective tissue structure built of specialized cells called chondrocytes and an extracellular matrix (ECM) that is normally rich in water (more than 70%) and also contains various organic components. These include collagen type II, glycosamino-glycans (chondroitin sulfate and keratan sulfate) and protein molecules such as decorin, aggrecan and fibromodulin. These ECM components are produced by chondrocytes and their synthesis is the subject of finely tuned regulation that involves various proteolytic enzymes [8,9,10]. The organization of the ECM is quite complex. A network of collagen type II fibrils is intertwined with proteoglycan aggregates made up of a ‘core protein’ (aggrecan) that is bound to glycosamino-glycans and further connected to a ‘backbone’ of hyaluronic acid [8,9]. The disruption of this complex organization marks the onset of OA. Two groups of proteolytic enzymes have been implicated in the degradation of the cartilage ECM. A Disintegrin and Metalloproteinase with Thrombospondin motifs (ADAMTS) is a family of enzymes responsible for the cleavage of aggrecan from the hyaluronic acid (in particular ADAMTS-4 and 5), while matrix metalloproteinases (MMPs), especially MMP-13, break down the collagen network [11,12]. These ECM alterations then lead to proliferation and hypertrophy of the chondrocytes whose ‘pro-synthetic’ phenotype is changed into a ‘pro-inflammatory’ one, leading to the production of potent mediators of inflammation such as interleukin 6 (IL-6), tumor necrosis factor-alpha (TNFα), interleukin 1β (IL-1β), which further stimulate the breakdown of the ECM, contribute to the occurrence of pain and activate the synovial membrane [8,13]. These pro-inflammatory mediators upregulate the production of ADAMTS, MMPs, cathepsins, cyclooxygenase-2 (COX-2) as well as reactive oxygen species (ROS) which can directly cleave collagen and aggrecan, thereby intensifying cartilage degradation [14]. The significance of proteolytic enzymes has also been demonstrated in animal studies, where knockout mice lacking ADAMTS-5 or MMP-13 did not develop experimental OA [15,16]. In addition to ECM degradation by ADAMTS and MMPs, the de novo synthesis of collagen type II and aggrecan is suppressed in chondrocytes of joints with OA [17].

A major molecular pathway implicated in the development of OA is the nuclear factor kappa-B (NF-kB) signaling pathway [18]. The nuclear translocation of NF-kB and its binding to regulatory segments in DNA initiates the cascade of cartilage degradation, as it leads to increased production of MMP-1, MMP-9 and MMP-13, ADAMTS-4 and 5 and secretion of the pro-inflammatory cytokines, which is then further stimulated through a positive feedback loop [19,20]. The mitogen-activated protein kinase (MAPK) pathway has been shown to upregulate the release of MMP-1 and MMP-13, increase collagen degradation and yield higher levels of TNFα and IL-1β through activation of regulatory gene p38 [21]. In addition, Wnt/β-catenin is another molecular pathway with a key role in OA. Upon binding to its receptor, Wnt leads to the activation of proteins which inhibit the cleavage of β-catenin and allow it to translocate into the nucleus. This is associated with an increase in MMP-13, ADAMTS-4 and 5 and reduced cartilage thickness [22]. Furthermore, Wnt/β-catenin inhibition has been shown to reduce cartilage degradation and ameliorate OA severity in a mouse model [23].

Articular cartilage degradation, however, is not the only hallmark of OA. Subchondral bone changes in the early stages of OA include remodeling of the subchondral plate, increased porosity, decreased thickness of the trabeculae and bone mineral density. Late-stage OA, on the other hand, is characterized by subchondral bone sclerosis with increased trabecular thickness [24,25]. In addition, subchondral bone cysts in weight-bearing segments of the joint have been described as containing a high number of osteoblasts, osteoclasts and osteoprogenitor cells, which indicates significant bone turnover [26]. These osteoblasts show stronger alkaline phosphatase activity and release higher levels of transforming growth factor β1 (TGFβ1), insulin-like growth factor 1 (IGF1), vascular endothelial growth factor (VEGF) and receptor activator of nuclear factor kappa beta-ligand (RANKL) compared to normal osteoblasts [25]. Synovial inflammation has been associated with pain, increased cartilage degradation and radiographic progression with narrowing of the joint space [8,27]. The synovium of OA joints is rich in macrophages, mast cells, T- and B-cells and the synovial fluid contains a variety of pro-inflammatory mediators, including TNFα, IL-6, IL-1β, TGFβ1, VEGF, leukotrienes and prostaglandins, which can stimulate the release of proteolytic enzymes by chondrocytes [27,28,29,30]. The role of synovial macrophages in particular has been widely investigated in OA. Their ability to recognize danger-associated molecular patterns (DAMPs) such as cartilage fragments and intracellular proteins from damaged cells leads to an increased secretion of pro-inflammatory cytokines [31]. Classically, two distinct phenotypes of synovial macrophages have been described. M1, or pro-inflammatory macrophages, have been associated with progression of OA through amplification of MMP-1 and 3 and ADAMTS-4 and 5 and downregulation of collagen and aggrecan synthesis. M2, or anti-inflammatory macrophages promote a ‘protective environment’ in the OA joint through secretion of IL-4 and 10 [31]. The M1/M2 ratio has been found to be markedly elevated in OA knees and correlates with radiographic progression [32]. Finally, pathological manifestations in Hoffa’s fat pad include an increase in vascularization and fibrosis, thickening of the interlobular septa, infiltration of inflammatory cells and higher levels of VEGF and IL-6 [33].

## 3. The Link between Obesity and Knee OA

While aging remains the primary cause for development of OA, overweight (defined as a body mass index (BMI) > 25 kg/m^2^) and obesity (defined as a BMI > 30 kg/m^2^) represent major risk factors associated with knee OA [2,3,34]. In fact, obesity in younger age groups (20–29 and 30–39) has been associated with a higher incidence of knee but not of hip OA, suggesting that the impact of obesity predates that of physiological aging [35]. In addition, higher pain scores, impaired mobility and lower physical activity have all been associated with increased BMI levels [3]. The higher mechanical load exerted on the knees as weight-bearing joints leads to impaired cartilage homeostasis [2]. Animal studies found this to be associated with reduced cartilage thickness, rapid degeneration, increased subchondral bone thickness and occurrence of bone marrow lesions [36,37]. Weight overloading of joints has been shown to upregulate the secretion of TNFα and IL-1β thus mediating the degradation of cartilage ECM [38]. Furthermore, the higher thigh girth seen in overweight patients changes the alignment of lower leg joints—greater abduction of the hip and varus deformity of the knee lead to predominant load transfer along the medial portion of the knee where articular cartilage suffers earlier damage [2]. While increased body weight leading to altered mechanical load on the knees can certainly account for some of the risk of OA development, obesity has also been implicated in OA of non-weight-bearing joints which hints at the role of its systemic effects [3]. Different measurements of increased body weight such as BMI, fat percentage, abdominal obesity and amount of visceral abdominal tissue have all been associated with development of hand OA, while higher BMI correlates with pain intensity in subjects with hand OA [38]. In fact, it has been proposed that obesity’s role in the development and progression of OA combines the impact of increased body weight and the establishment of a chronic ‘micro-inflammatory state’ [39]. Therefore, obesity-related OA can be categorized as a separate type of secondary OA, different from aging-associated primary OA and other cases of OA secondary to trauma or inflammatory arthritis.

Recent studies have elaborated on the role of adipose tissue as a potential source of cytokines, chemokines and other mediators, collectively known as adipokines [40]. These mediators promote either cartilage degradation or the preservation of its integrity through complex interactions (Table 1) [38,40]. In addition, obesity leads to a change in the cellular profile of resident macrophages, from the anti-inflammatory M2 phenotype to the pro-inflammatory M1 phenotype, which stimulates the release of pro-inflammatory cytokines such as TNFα, IL-6 and IL-1β [41].

One major adipokine implicated in the development of knee OA with an apparently contrasting impact is adiponectin. Higher serum levels of adiponectin have been associated with the formation of osteophytes, narrowing of the joint space and higher radiographic score [42]. On the other hand, adiponectin appears to have anti-inflammatory properties by altering the phenotype of resident adipose tissue macrophages from M1 to M2 and by decreasing the levels of TNFα [43]. Moreover, adiponectin has been shown to lower serum levels of triglycerides and free fatty acids, thereby reducing the level of oxidative stress [44]. At the joint level, adiponectin upregulates the expression of the tissue inhibitor of MMP-2 (TIMP-2), which also counters the action of ADAMTS and inhibits the IL-1β-stimulated expression of MMP-13 [44,45].

The serum levels of another major adipokine, leptin, are also increased in overweight patients [46]. They correlate with a higher BMI, a higher Western Ontario and McMaster Universities Arthritis Index (WOMAC) pain score and structural damage [47]. On the other hand, the decrease of serum leptin seen in weight loss has been associated with the improvement of patients’ symptoms [46]. Chondrocytes from OA joints have also demonstrated higher leptin levels and this local increase in the concentration of this particular adipokine upregulates a plethora of pro-inflammatory cytokines such as IL-6, IL-1β, vascular cell adhesion molecule-1 (VCAM-1) and prostaglandin E2, as well as multiple MMP subtypes (namely MMP-1, 3, 9 and 13) by chondrocytes, which promote cartilage degradation [48,49]. In addition, leptin stimulates synovial fibroblasts to release IL-6 and IL-8 and mediates subchondral bone remodeling by increasing the alkaline phosphatase activity of osteoblasts, where its expression is higher than that of normal osteoblasts [50].

Resistin, an adipokine associated with the progression and severity of knee OA, drives the production of MMPs and pro-inflammatory cytokines by chondrocytes and promotes structural damage [51,52,53]. Its serum levels are higher in OA patients compared to healthy controls and correlate with the development of cartilage defects and bone marrow lesions, which are associated with higher pain score and more pronounced structural alterations [53]. Visfatin is a pro-inflammatory adipokine implicated in the pathogenesis of OA through its upregulation of TNFα, IL-6, IL-1β, MMP-3 and 13 and ADAMTS-4 and 5. It is among the major promoters of cartilage degradation by inhibiting the synthesis of collagen type II and proteoglycans with high molecular weight. Serum and synovial fluid levels of visfatin show a positive correlation with structural damage, markers indicating degradation of collagen type II and aggrecan, CRP levels and OA symptoms [54]. 

Dyslipidemia, another major aspect of obesity, as well as the associated lipotoxicity have recently been implicated in OA [55,56]. Altered lipid metabolism and an upregulation of two key enzymes—25-hydroxycholesterol 7α-hydroxylase and cholesterol 25-hydroxylase—have been reported in OA and likely play a key role in structural damage through the promotion of MMP and ADAMTS. This, in turn, has been associated with structural changes such as synovitis, formation of osteophytes and subchondral bone sclerosis [57]. Cartilage degradation is also worsened through the action of reactive oxygen species (ROS) and free fatty acids which induce mitochondrial dysfunction in chondrocytes and stimulate the release of pro-inflammatory cytokines [58,59,60]. A recent cross-sectional study found higher levels of total cholesterol, triglycerides and low-density lipoprotein (LDL) in patients with symptomatic knee OA [61]. These data confirmed earlier observations that hypertriglyceridemia and decreased high-density lipoprotein (HDL) are associated with more severe knee pain [62]. In addition, higher levels of LDL provoke synovial inflammation and ectopic bone formation by stimulating synovial cells and are associated with higher knee pain [55].

Age-related muscle tissue loss and dysfunction also play a key role in the development of knee OA. However, it is a special type of body composition entity, known as sarcopenic obesity, which has recently been studied in the context of knee OA and appears to be more tightly associated with it than non-sarcopenic obesity [63]. It refers to cases in which the increase in body weight caused by obesity is compensated for by a loss of muscle mass [63]. Sarcopenic obesity has a complex pathogenesis which is influenced by age-related changes in physical activity, nutrition and hormonal profile (such as altered secretion of insulin, parathyroid hormone, sex hormones and vitamin D) [63]. Adipokines, as mediators of systemic chronic inflammation, are another factor behind progressive muscle loss. The serum levels of leptin are negatively correlated with skeletal muscle mass and are increased in subjects with sarcopenic obesity [64]. This is thought to be a result of decreased number of leptin receptors and development of leptin resistance, which in turn causes elevated systemic levels of TNFα, IL-6 and promotes insulin resistance [64]. Conversely, high adiponectin levels have been reported in the serum of sarcopenic subjects as a compensatory mechanism aimed at countering the breakdown of their muscle proteins [65]. Furthermore, adiponectin levels are low in obese patients and subjects with insulin resistance [64]. This suggests that obese patients who subsequently develop sarcopenia would have low serum adiponectin levels and thus be unable to compensate for muscle loss. Sarcopenia has recently been found in as many as 45.2% of cases of knee OA, which is twice as high as in healthy controls [66]. A possible pathogenic mechanism linking the two entities could be an impaired afferent input from the OA knee leading to an altered efferent motor neuron impact on the quadriceps [66]. The latter causes quadricep inhibition and weakness and is a separate risk factor for knee OA progression and OA-related pain.

## 4. Pain in Knee OA

While the hallmark of OA is the degradation of articular cartilage, blood vessels and nerve endings are not present inside the cartilage and so it cannot generate pain sensation directly [67,68]. However, the synovium, joint capsule, adjacent ligaments, subchondral bone and Hoffa’s fat pad are all richly innervated by sensory and sympathetic nerve fibers and can produce nociceptive pain. Nociceptor nerve terminals register stimuli of mechanic, chemical or thermal origin and release an array of neuropeptides, including substance P (SP) and calcitonin gene-related peptide (CGRP) [69]. The pain signal is then transmitted along the dorsal root ganglia to the posterior horn of the spinal cord and the bodies of the sensory neurons, leading to an upregulation of the NOD-like receptor (NLR) family pyrin domain—containing protein 3 (NLRP3) inflammasome, C-C motif chemokine ligand 2 (CCL2) and its receptor (CCR2) and the Wnt/β-catenin pathway [68]. Additional local release of SP and CGRP recruits second-order neurons through which the pain stimulus is ultimately conveyed via ascending pathways to the central nervous system (CNS) where the conscious awareness of pain takes place [70,71,72]. 

Apart from nociceptive pain arising from damaged tissue, knee pain in OA may also be driven by neuropathic pathways [68]. Cytokines such as TNFα, IL-6, IL-1β and nerve growth factor (NGF) and chemokines such as CCL2 are implicated not only in the occurrence of nociceptive pain but also in the peripheral sensitization of sensory nerve fibers [73]. This persistence of the pain stimulus ultimately causes central sensitization leading to hyperalgesia and tenderness around the joint, which is typical of neuropathic pain [70,71]. These findings are supported by the fact that patients with less severe joint alterations may experience debilitating persistent joint pain [74]. In addition, pain intensity and central sensitization in OA patients were positively correlated with NGF levels in the synovial fluid [75]. While OA pain is usually located at the site of the affected joint and elicited during weight bearing, exercise, walking and climbing or descending stairs and is relieved at rest, increased central sensitization leading to neuropathic pain may be associated with pain at rest, poorly located pain around the joint structures and postoperative pain persistence following total joint arthroplasty [68,75,76]. Multiple pain mediators including growth factors, cytokines and chemokines have been studied in the context of both nociceptive and neuropathic pathways in OA and are reviewed below.

NGF is a primary cytokine regulating pain stimulus transduction which is upregulated in OA joints through increased mechanical stress and the action of IL-1β TNFα and visfatin, which promote its synthesis by synoviocytes, chondrocytes, macrophages, mast cells and neutrophils [77,78]. NGF acts on tropomyosin receptor kinase A (TrkA) which leads to the upregulation of an array of inflammatory cytokines, as well as neurotransmitters such as SP and CGRP, which are involved both in peripheral and central pain mechanisms. Knee OA models based on destabilization of the medial meniscus have been associated with higher expression of NGF and pain-related behavioral changes in mice [79]. A recent study on a model of knee OA induced by medial meniscectomy found that NGF expression in the synovium increased in early OA and subsequently decreased in advanced stages but continued to increase in osteochondral channels and bone marrow [80]. These changes were mirrored by similar time- and site-specific increases in CGRP-mediated sensory innervation [80]. Consequently, a number of clinical trials attempted to use anti-NGF antibodies for treatment of OA but results of rapidly progressive cartilage degradation halted these investigations. These therapeutic interventions will be discussed further below.

The generation and maintenance of pain patterns in OA including mechanical allodynia and movement-evoked pain are associated with molecular changes in sensory neurons of the dorsal root ganglia, including chemokines and their receptors [68]. CCL2 and its interaction with receptor CCR2 has recently been implicated in the development of knee OA pain through local accumulation of macrophages and monocytes [81]. On the other hand, blockade of CCL2/CCR2 signaling led to a decrease in macrophage recruitment and ameliorated the severity of knee synovitis and cartilage lesions in a mouse model of OA [82]. The intra-articular injection of recombinant CCL2 caused knee hyperalgesia by directly stimulating sensory neurons rich in CCR2. Conversely, the administration of a receptor antagonist resolved the established hyperalgesia [83]. These data suggest that CCL2 acts both through local recruitment of inflammatory cells and subsequent action of mediators on nociceptors, as well as through direct activation of sensory neurons and upregulated afferent pain transduction.

CGRP is a neuropeptide largely found in nociceptive neurons in the dorsal root ganglia and in nerve fibers reaching the posterior horn of the spinal cord [68]. In addition, CGRP acts upon synoviocytes, endothelial and inflammatory cells, thus influencing local angiogenesis and inflammation. Elevated levels of CGRP have been found in the serum, synovial fluid and synovial tissue of OA patients and have been correlated with pain intensity [84]. Moreover, CGRP-containing nerve fibers can directly regulate bone remodeling through promotion of osteogenesis and inhibition of osteoclastogenesis [84]. These findings are supported by the established association between serum and synovial fluid levels of CGRP and osteophyte formation in the knee as a whole and in the medial compartment alone [85]. In addition, as OA progresses, sensory nerve terminals rich in CGRP grow along newly formed blood vessels into channels that penetrate articular cartilage [86]. Therefore, joint structures that are normally not innervated might subsequently become sources of pain [87]. Nevertheless, the precise role of CGRP in OA pain remains to be fully elucidated.

As outlined above, pro-inflammatory cytokines such as TNF-α, IL-1β and IL-6 have been well studied in the pathogenesis of OA, however their role in pain mechanisms remain controversial. Notably, clinical trials of anti-cytokine medication in OA patients did not lead to satisfactory pain relief [68]. An earlier study found no correlation between IL-6 levels and WOMAC pain score [88]. This was later challenged by other authors who established a statistically significant positive correlation between IL-6 levels in the synovial fluid and WOMAC pain score [89]. One study found that while synovial levels of TNFα correlated with total WOMAC score, pain during movement and while at rest, synovial levels of IL-1β were inversely associated with knee pain [90]. Injection of TNF induced persistent sensitization of peripheral sensory neurons in the knee, assessed through development of mechanical allodynia. The concomitant administration of TNFα-inhibitor etanercept or a COX-inhibitor reversed that development [91]. It was later reported that IL-1β and IL-6 were negatively correlated with knee pain score and Kellgren-Lawrence score and hypothesized that pain in the early stage of knee OA may be of inflammatory origin and driven by the above cytokines. Conversely, in late-stage OA other sources of pain rather than inflammation need to be investigated [92]. These observations are further supported by an earlier report which found that levels of TNFα and IL-6 were significantly higher in early as opposed to late-stage knee OA [93]. IL-1β has been shown to increase the production of proteolytic enzymes by chondrocytes and to promote osteoclast differentiation [94]. In addition, it is directly involved in pain genesis by upregulating the expression of NGF [95]. A recent paper on an experimental model of knee OA found that a deficiency in type 1 interleukin-1 receptor (IL-1R1) did not alter the course of cartilage degradation but prevented the occurrence of pain [96]. The NLRP3 inflammasome is part of the innate immune system which regulates active IL-1β [68]. As previously demonstrated, the activation of the inflammasome leads to an upregulation of IL-1β, followed by the release of other pro-inflammatory cytokines and the synthesis of proteolytic enzymes such as MMP-13 and ADAMTS5, which directly cause synovial inflammation and cartilage degradation [97]. On the other hand, OA-related risk factors such as oxidized LDL, elevated cholesterol, hydroxyapatite crystals and others are recognized as DAMPs which trigger the NLRP3 pathway and lead to a specialized type of programmed cell death of the chondrocytes, known as pyroptosis [98]. These pyroptotic chondrocytes release IL-1β and TNFα, which in turn promote the release of MMPs and ADAMTS causing further cartilage damage in a vicious cycle. In addition, the crosstalk between neutrophils and macrophages may also be implicated. Neutrophil extracellular traps (NETs) may prime macrophages and promote the activation of the NLRP3 inflammasome, which upregulates IL-1β and IL-18 that further stimulate neutrophils to form NETs—A cascade which has been recently studied in inflammatory conditions such as atherosclerosis and rheumatoid arthritis [99,100]. Since inflammasomes are involved in the low-grade inflammation seen in obesity and metabolic syndrome, they are likely key structures in the development of obesity-related OA. The inhibition of the NLRP3 inflammasome in a rat model of knee OA via an intra-articular injection of the anti-inflammatory molecule dexmedetomidine improved pain and cartilage tissue damage, which was associated with lower levels of TNFα, IL-1β, IL-6, MMP-13 and an upregulation of collagen type II [101].

These data underscore the complexity of pain generation and maintenance in OA and provide a platform for further studies into pain mechanisms in obesity-related knee OA. As outlined above, obesity-related knee OA is associated with higher levels of the pro-inflammatory cytokines (and, therefore, with increased joint pain and structural damage) and an altered adipokine profile. To the best of our knowledge, the link between adipokines and pain at the molecular level has only been studied in a 2014 paper, where the authors found that visfatin increases the synthesis of NGF and can modulate pain occurrence [77]. Clinical outcome-based studies identified that a higher degree of obesity is associated with a higher pain and disability score [3]. An imaging-based study found that the occurrence of bone marrow lesions on magnetic resonance imaging (MRI) was associated with various components of dyslipidemia, including hypercholesterolemia, hypertriglyceridemia and low levels of high-density lipoproteins (HDL) [102]. A recent paper by Valdes attempted to ‘connect the dots’ between obesity and OA pain on the molecular level [103]. The transient receptor potential cation channel vanilloid subfamily member 1 (TRPV1) is involved in the modulation of nociceptive pain afferents to the spinal cord and has been associated with OA pain. TRPV1 knockout mice did not develop hypertension and impaired glucose tolerance and did not have an increased nociception when placed on a high-fat diet compared to controlled wild-type mice on the same diet, thus suggesting a common molecular pathway between OA and metabolic syndrome [104]. The action of some peripheral endogenous cannabinoids appears to provide a second link between OA and obesity. These mediators control appetite and energy metabolism and can reduce pain by acting on TRPV1 [105]. Cannabinoid receptor antagonists with peripheral action reduce body weight and improve dyslipidemia and insulin resistance and may prove beneficial as analgesics in OA [106]. The gut microbiome and its function provide another interesting association between metabolic syndrome and pain in OA. It has recently been demonstrated that gut microbiome dysbiosis is associated with OA pain and structural damage [107]. On the other hand, modulation of the gut microbiome via a higher dietary fiber intake is associated with lower body weight, total cholesterol and systolic blood pressure and protects against metabolic syndrome, coronary artery disease and diabetes [103]. In addition, an experimental study in mice revealed that obesity can itself modulate the gut microbiome by increasing the number of key pro-inflammatory species, which then drive the onset of systemic inflammation, accompanied by macrophage recruitment to the synovium and progression of OA damage [108]. These common molecular mechanisms, however, merit further investigation in future studies.

## 5. Therapeutic Approaches in Knee OA with Possible Benefit in Obesity-Related Knee OA

While recent years have seen an abundant amount of research into the pathogenesis of obesity-related OA, no specific therapeutic guidelines have been published [6]. The American College of Rheumatology (ACR) and the Osteoarthritis Research Society International (OARSI) have regularly updated their guidelines for the management of knee OA in general and they should be applied to obesity-related OA as well (Table 2) [109,110]. Nevertheless, before deciding on a particular therapeutic approach one should consider any possible adverse outcomes stemming from other possible obesity-associated conditions such as hypertension, insulin resistance or diabetes and dyslipidemia, to name a few.

Both ACR and OARSI guidelines strongly recommend exercise and weight loss which are of particular importance in subjects with obesity-related knee OA. Weight loss of ≥5% has been associated with better functional improvement and less pain intensity [109]. Another study found that losing between 10 and 20% of baseline body weight had a better impact on clinical and mechanical outcome as opposed to weight loss of <10%, and the results were statistically significant [111]. A randomized control trial of 89 patients with obesity and knee OA found that subjects who had adhered to a low-calorie diet and were frequently followed by a dietician achieved a mean weight loss of 10.9 kg and a statistically significant decrease in the WOMAC pain score [112]. Furthermore, weight loss decreases the impact of mechanical load on the knee and strategies to achieve it are a major element of efforts to slow down knee OA progression [2]. Literature data suggest that weight loss is associated with structural benefits in the knee such as increased proteoglycan content in the ECM and reduction in cartilage thickness loss, as well as increased serum levels of cartilage synthesis markers and decreased serum levels of cartilage degradation markers [113,114]. In one randomized control trial, 289 obese subjects were instructed to follow an exercise program aimed at strengthening the quadriceps over a period of two years, which was associated with a statistically significant decrease in knee pain [115]. Resistance training is also important in promoting muscle strength which improves joint mobility and reduces pain in 50–70% of patients with obesity-related knee OA [2]. Finally, exercise and weight loss are pivotal in the management of traditional cardiovascular risk factors associated with obesity [6]. 

Oral non-steroidal anti-inflammatory drugs (NSAIDs) and intra-articular injections of corticosteroids (CSs) are strongly recommended in knee OA in the general population. However, such drugs should be used with caution in patients with obesity-related knee OA who have a greater cardiovascular risk due to comorbidities associated with metabolic syndrome [6]. It is therefore advisable to prefer topical over oral NSAIDs in these patients, considering their equivalent therapeutic effect but more favorable safety profile due to a much slower absorption [116]. Intra-articular injections of either hyaluronic acid or CSs have been universally used with a favourable therapeutic outcome and few adverse effects. ACR’s guidelines, however, while strongly recommending the use of intra-articular CSs, do not include a statement on the use of hyaluronic acid [109]. OARSI’s guidelines, on the other hand, suggest the use of intra-articular injections of CSs as a means to provide short-term pain relief, whereas injections of hyaluronic acid are recommended for pain relief over a longer period (12 weeks and beyond) and are considered a safer option compared to repeated CS injections [110]. However, one must consider that the effect of intra-articular injections is lower in patients with higher BMI [6]. The use of anti-obesity drugs has been suggested for patients with BMI > 30 but has not yet been fully studied in associated knee OA [117].

An interesting therapeutic approach has recently been suggested based on the molecular subtype of knee OA and identified pathogenic mechanisms [118]. The authors distinguished between four separate subtypes of progressive knee OA, each represented by the presence of distinct molecular profiles, summarized in Table 3. In addition, they suggested pathogenesis-based preferential treatment options, which are indicated in Table 4.

Considering that obesity-related knee OA shares features of all the above molecular subtypes, it could be difficult to point to a single therapeutic approach. A comprehensive literature review on the repeated intra-articular administration of hyaluronic acid at 6-month-long intervals found pain reduction from baseline and sustained or further reduced pain over the course of repeated injections, with the longest follow up period being 25 months [119]. A post-hoc analysis of the prospective, double-blind, randomized, multicenter and parallel-group ‘HAV-2012 trial’ compared pain and function scores before and six months after intra-articular viscosupplementation, and juxtaposed the results with weight status (obese vs. non-obese) and structural changes (assessed through mild, moderate or severe radiological severity based on OARSI’s 1, 2 or 3 grade) [120]. The study found that WOMAC pain score decreased significantly in all patient subgroups—obese, OARSI grade 3, obese and OARSI grade 3, neither obese nor OARSI grade 3. However, the WOMAC pain score was significantly lower in non-obese versus obese patients and in patients with knee OA OARSI grade 1 or 2 versus grade 3. Still, in patients who responded to treatment, the pain score at baseline and its subsequent decease were not related to weight status and radiological severity. Therefore, the authors concluded that while it was less likely for obese patients to respond to treatment, for those reporting benefit from the therapy, pain relief would be similar to that in non-obese patients [120]. Based on these findings, we would suggest that the intra-articular administration of hyaluronic acid could be recommended in obese patients. However, if no clinical benefit (i.e., pain relief, improved mobility) is reported by the patient at six months from baseline, then repeated injections would not be advisable. A recent meta-analysis on a total of 3793 patients, 1067 of whom received a combination of glucosamine and chondroitin as treatment for knee OA, found a statistically significant improvement in the total WOMAC score and the joint space narrowing in the combination group, but no difference in the pain score assessed on a visual analog scale (VAS) [121]. Considering the need to avoid NSAIDs and CSs in patients with obesity-related knee OA, glucosamine, chondroitin and some natural antioxidants such as curcumin, oleuropein and ginger extracts, may be preferred owing to their favorable benefit/risk ratio and potential positive impact on both metabolic syndrome and OA [6]. Experimental animal studies with curcumin showed a marked chondroprotective role via suppression of aggrecan degradation, inhibition of MMP-3, 8 and 13 and ADAMTS-5 and upregulation of the synthesis of collagen type II [122,123,124]. Oleuropein, a potent antioxidant extracted from olive tree leaves and found in olive oil, has anti-obesity effects and lowers systemic inflammation, blood pressure and cholesterol levels [125]. In a study on human chondrocytes, oleuropein downregulated the IL-1β-induced activation of the NFκB pathway and reduced the levels of MMP-1, MMP-13, and ADAMTS5, thus countering cartilage matrix degradation [126]. However, a recent randomized control trial on 124 subjects with knee pain taking oleuropein as a dietary supplement on a daily basis for 6 months showed no difference to placebo with regard to serum levels of inflammatory and cartilage remodeling biomarkers and a significant effect was only reported in a subgroup of patients with severe walking pain at baseline [127].

As indicated in Table 4, bisphosphonates may have beneficial effect in knee OA of the bone-remodeling subtype. One meta-analysis of seven randomized, placebo-controlled trials of bisphosphonates in knee OA did not show an improvement in either symptoms or radiographic progression [128]. However, the authors suggested that bisphosphonates may be potentially beneficial in patients with high subchondral bone turnover. A recent randomized, double-blinded, placebo-controlled trial of zoledronic acid in 223 participants with knee OA, which used bone marrow lesions seen on MRI as evidence of subchondral bone remodeling, found that an early infusion of zoledronic acid did not reduce knee pain or the size of bone marrow lesions significantly over 24 months [129]. In addition, it has been reported that bisphosphonates may slow down radiographic progression, but their results were significant only in non-overweight patients with mild radiographic damage at baseline [130]. Their secondary analysis in obese patients found no significant effect [130]. These data show that the use of bisphosphonates in knee OA is likely not sufficiently beneficial and there is currently not enough evidence to support their use in cases of obesity-related knee OA. The use of calcitonin was also refuted by two randomized, double-blind, multi-center, placebo-controlled trials, which found no effect on joint space narrowing and non-significant effect on the total WOMAC score [131]. 

While initially considered especially promising in treating chronic OA pain, NGF inhibitors demonstrated considerable side effects including osteonecrosis and progressive cartilage damage, leading to a temporary suspension of clinical trials, which were resumed in 2012 [68,132]. Currently, lower doses of the anti-NGF monoclonal antibody tanezumab (2.5 to 5 mg, administered subcutaneously) have shown a statistically significant improvement in WOMAC pain and WOMAC function score, while having a favorable safety profile and an occurrence of rapidly-progressive OA in only 1.4–2.8% of subjects [133]. Another strategy to limit the adverse effects of NGF blockade could be to target its receptor, TrKA. A significant reduction in WOMAC pain and functional benefits has been reported in patients with painful knee OA following intra-articular administration of a TrKA inhibitor and an acceptable safety profile, with transient and self-limited reactions at the injection site [134]. Another novel therapeutic strategy targets TRPV-1 through intra-articular injections of capsaicin and resiniferatoxin (RTX). Highly purified synthetic trans-capsaicin, which specifically targets TRPV1-containing pain nociceptors while sparing sensory fibers for touch or pressure, has shown a rapid and significant impact on WOMAC walking pain scores and a favorable safety profile [135,136]. An injectable form of RTX has recently been developed and given a ‘breakthrough therapy’ status by the United States Food and Drug Administration (FDA) [137]. It is currently being investigated for the treatment of knee OA pain in a Phase III clinical trial but preliminary results from the Phase IB trial showed that 83% of patients had a statistically significant, clinically meaningful decrease in the WOMAC walking pain score [138]. Side effects included injection site pain, nausea, vomiting and headache but were manageable and well tolerated [138]. Despite these promising data, a separate analysis of these novel molecules in obesity-related knee OA is still lacking.

Anti-cytokines have long been studied as a possible disease-modifying treatment in OA, however results remain controversial. An open-label randomized control trial comparing the intra-articular administration of TNFα-inhibitor adalimumab versus hyaluronic acid in patients with moderate to severe knee OA reported that pain improvement on VAS and decrease of the WOMAC pain score were significantly greater in the adalimumab group [139]. A similar study of etanercept versus hyaluronic acid in moderate to severe knee OA also found a greater decrease in pain on VAS at post-injection weeks one and two and in the WOMAC pain score on post-injection week four in the group treated with the TNFα-inhibitor [140]. Conversely, the recent OKINADA randomized clinical trial on the efficacy and safety of adalimumab in inflammatory OA of the knee found no significant improvement in pain and joint function in patients with established radiographic knee OA [141]. A novel drug currently under investigation in patients with painful knee OA is the monoclonal antibody MEDI7352, which binds specifically to NGF and TNFα, thus blocking both mediators of pain and inflammation [68]. Autologous conditioned serum (ACS) is another therapeutic option with pain-modifying and anti-inflammatory properties, mainly mediated by the blockade of IL-1′s action via the IL-1 receptor antagonist (IL-1Ra). It is derived from the patient’s own blood and is rich in cytokines and growth factors secreted by platelets upon stimulation with glass beads [142]. A crucial step in the preparation is the incubation time, varying anywhere between 1 and 24 h, however a recent study found that incubation for 3 h had the optimal ratio between anti- to pro-inflammatory cytokines, with higher IL-1Ra levels and lower TNFα levels [143]. In addition, levels of IL-1Ra and platelet-derived growth factor (PDGF) were significantly higher in ACS than in platelet-rich plasma (PRP), suggesting better clinical efficacy [143]. Other authors compared the efficacy of intra-articular administration of ACS, PRP, hyaluronic acid and GCs and found that both blood-derived products performed better in terms of pain relief and functional improvement without a statistically significant difference between them [144]. Other studies, however, showed higher pain reduction and functional improvement with ACS compared to PRP, likely due to the higher IL-1Ra levels, decrease in IL-1β and improved synovial fluid viscosity [145,146]. Furthermore, it has been found that 67% of subjects with painful knee OA who had failed previous standard therapy, including physiotherapy and PRP, had a reduction of pain assessed on VAS one month after administration of ACS and this result persisted at 6 and 12 months of follow-up [147]. A notable finding from this study was that responders had significantly higher levels of IL-1Ra in their ACS compared to non-responders, underscoring the potential benefits of intra-articular IL-1β blockade in knee OA [147]. These reports suggest that anti-cytokine therapy could have potential benefits in obesity-related knee OA considering their role in maintaining the low-grade inflammatory state characteristic of obesity, however they are yet to be studied in such conditions. Finally, anti-obesity drugs have been proposed in patients with BMI > 30 and associated metabolic disorders, but their role in the therapy of concomitant knee OA requires further investigation [47].

## 6. Conclusions

Obesity-related knee OA is a separate entity that is not simply related to increased mechanical load but represents a disorder with complex pathogenesis driven by the chronic micro-inflammatory state, altered adipokine profile and sarcopenia seen in obese individuals. It is associated with higher pain score, decreased joint functionality and poorer quality of life. While recent years have seen a spike in research in that field, much remains to be elucidated. Future studies should focus on possible correlations between serum and synovial levels of adipokines such as leptin, resistin and adiponectin and mediators such as NGF and CGRP in order to address the molecular basis of already established clinical associations between their expression and pain severity. These could be essential in developing better pain management strategies. While current treatment guidelines do not feature specific recommendations for obesity-related knee OA, there is sufficient evidence to support exercise and weight loss. Viscosupplementation, glucosamine, chondroitin and natural antioxidants may also have potential benefits, whereas oral NSAIDs and GC injections should be avoided due to increased cardiovascular risk. Novel therapeutic approaches including intra-articular administration of inhibitors of key pain mediators such as NGF and TRPV-1 have yielded promising early results but are yet to be studied in the context of obesity.

## Figures and Tables

**Table 1:** Major adipokines implicated in obesity-related knee OA and their impact.

Adipokine	Proposed Role in Knee OA
Adiponectin	Protective; decreases the levels of TNFα; lowers IL-1β-stimulated expression of MMP-13; promotes TIMP-2 which inhibits MMP-2 and ADAMTS.
Leptin	Harmful; upregulates IL-6, IL-1β, prostaglandin E2, MMP-1, 3, 9 and 13; mediates subchondral bone remodeling; correlates with higher pain score.
Resistin	Harmful; upregulates MMPs and pro-inflammatory cytokines; correlates with development of cartilage and bone marrow lesions and higher pain score.
Visfatin	Harmful; upregulates TNFα, IL-6, IL-1β, MMP-3 and 13 and ADAMTS-4 and 5; correlates with structural damage and OA symptoms.

ADAMTS–A Disintegrin and Metalloproteinase with Thrombospondin motifs; IL-1β–interleukin-1 beta; IL-6–interleukin-6; MMP–matrix metalloproteinase; TIMP–tissue inhibitor of matrix metalloproteinase-2; TNFα–tumor necrosis factor alpha.

**Table 2:** ACR and OARSI guidelines on the management of knee osteoarthritis by strength of recommendation [109,110].

Type of Recommendation	ACR	OARSI
Strongly recommended	Exercise Self-efficacy and self-management programs Weight loss Cane Tibiofemoral knee brace Tai Chi Oral and topical NSAIDs Intraarticular CSs	Core recommendation–Arthritis Education; Structured Land-Based Exercise Programs (Type 1–strengthening and/or cardio and/or balance training/neuromuscular exercise OR Type 2–Mind-body Exercise including Tai Chi or Yoga) with or without Dietary Weight Management Topical NSAIDs
Conditionally recommended	Heat, Therapeutic cooling Cognitive behavioral therapy Acupuncture Kinesiotaping Balance training Patellofemoral knee brace Yoga Acetaminophen Tramadol Duloxetine Topical capsaicin	Aquatic exercise Gait aids Self-management programs Cognitive behavioral therapy with exercise Non-selective NSAIDs with or without PPIs (excluding patients with GI or CV comorbidities) COX-2 inhibitors (excluding patients with CV comorbidities) Intraarticular CSs Intraarticular hyaluronic acid

COX-2–cyclooxygenase-2; CSs–corticosteroids; CV–cardiovascular; GI–gastrointestinal; NSAIDs–non-steroidal anti-inflammatory drugs; PPIs–proton pump inhibitors.

**Table 3:** Molecular subtypes of knee osteoarthritis and key mediators [118].

Knee Osteoarthritis Subtype	Representative Molecules
Cartilage degradation-driven subtype	CTX-II, C2C, C2M, C-Col X
Bone remodeling-driven subtype	ALP, CTX-I, C1M, NTX-I
Pain-driven subtype	Bradykinin, CGRP, hs-CRP, NGF
Inflammation-driven subtype	IL-1β, IL-1Ra, IL-6, TNFα

ALP–alkaline phosphatase; CGRP–calcitonin gene-related peptide; CTX-I–C-telopeptide fragments of collagen type I; CTX-II–C-telopeptide fragments of collagen type II; C2C–cleavage neoepitope of collagen type II; C1M–fragments of collagen type I degraded my matrix metalloproteinases; C2M–fragments of collagen type II degraded my matrix metalloproteinases; C-Col X–C-terminus of collagen type X; hs-CRP–high sensitive C-reactive protein; IL-1β–interleukin-1 beta; IL-1Ra–interleukin-1 receptor antagonist; IL-6–interleukin-6; NGF–nerve growth factor; NTX-I–N-telopeptide of collagen type I; TNFα–tumor necrosis factor alpha.

**Table 4:** Potential pathogenesis-based preferential therapeutic approaches based on molecular subtype of knee osteoarthritis [118].

Knee Osteoarthritis Subtype	Treatment Principle	Potential Beneficial Therapies
Cartilage degradation-driven subtype	Supplementation of cartilage extracellular matrix components	Hyaluronic acid, chondroitin, glucosamine, undenatured collagen type II
Bone remodeling-driven subtype	Anti-bone resorption	Bisphosphonates, calcitonin, osteoprotegerin
Pain-driven subtype	Analgesia, anti-inflammation	NSAIDs, opioids, CGRP inhibitor, NGF inhibitor, capsaicin
Inflammation-driven subtype	Anti-inflammation	TNFα-inhibitor, IL-1-inhibitor, IL-1Ra, NSAIDs, COX-2-inhibitor

CGRP–calcitonin gene-related peptide; COX-2–cyclooxygenase 2; IL-1–interleukin-1; IL-1Ra–interleukin-1 receptor antagonist; NSAIDs–non-steroidal anti-inflammatory drugs; NGF–nerve growth factor.
